# Porous Bilayer Vascular Grafts Fabricated from Electrospinning of the Recombinant Human Collagen (RHC) Peptide-Based Blend

**DOI:** 10.3390/polym13224042

**Published:** 2021-11-22

**Authors:** Thi My Do, Yang Yang, Aipeng Deng

**Affiliations:** 1 School of Environmental and Biological Engineering, Nanjing University of Science and Technology, Nanjing 210094, China; Domy0207cnsh@gmail.com (T.M.D.); Aipengd@njust.edu.cn (A.D.); 2 Biomanufacturing Center, Department of Mechanical Engineering, Tsinghua University, Beijing 100084, China

**Keywords:** electrospinning, recombinant human collagen (RHC), bilayer vascular grafts, tissue engineering

## Abstract

Cardiovascular diseases, including coronary artery and peripheral vascular pathologies, are leading causes of mortality. As an alternative to autografts, prosthetic grafts have been developed to reduce the death rate. This study presents the development and characterization of bilayer vascular grafts with appropriate structural and biocompatibility properties. A polymer blend of recombinant human collagen (RHC) peptides and polycaprolactone (PCL) was used to build the inner layer of the graft by electrospinning and co-electrospinning the water-soluble polyethylene oxide (PEO) as sacrificial material together with PCL to generate the porous outer layer. The mechanical test demonstrated the bilayer scaffold’s appropriate mechanical properties as compared with the native vascular structure. Human umbilical vein endothelial cells (HUVEC) showed enhanced adhesion to the lumen after seeding on nanoscale fibers. Meanwhile, by enhancing the porosity of the microfibrous outer layer through the removal of PEO fibers, rat smooth muscle cells (A7r5) could proliferate and infiltrate the porous layer easily.

## 1. Introduction

Cardiovascular diseases are currently the leading causes of death worldwide [1,2,3]. Nearly 250,000 open surgical revascularizations are performed each year for diseased peripheral and coronary arteries in the United States alone [4,5]. Almost 23.3 million people are anticipated to die from coronary heart disease and heart attacks by 2030 [3]. While native vein and artery sections remain the best way to repair defected blood vessels via peripheral coronary bypass procedures, their availability is limited when the autologous blood vessels are occluded or diseased, or when their size does not correspond with the defected side [6]. The suitable native vein is unavailable among 30% of patients, thus, prosthetic vascular grafts with biocompatible biomaterials are highly demanded [7]. Designing multi-layered vascular scaffolds is considered an effective approach to mimic the structure and function of native blood vessels. In particular, electrospinning is one of the useful methods to obtain the vascular scaffolds since it is easier to develop multi-layered scaffolds by blending or mixing various materials and building non-delamination layers [8,9,10]. The electrospun technique uses high-voltage electrostatic fields to generate fibrous structures, which provide a biomimetic cellular environment resembling the extracellular matrix (ECM) of native blood vessels [11,12,13,14]. Electrospinning allows for fabrication of nano- to micro-scale fibrous matrices and for control of the composition, structure, and biomechanical properties of scaffolds [15,16]. This technique fabricates fibrous matrices from various natural polymers like collagen [17,18,19] and fibrin [20], as well as a wide variety of synthetic polymers like poly-ε-caprolactone (PCL) [19,21], poly (L-lactide) (PLLA) [22], and poly (D-lactide) (PDLA) [23], in addition to copolymers poly (L-lactide caprolactone) (P(LLA-CL) [24] and poly (D,L-lactide-co-glycolide) (PLGA) [25,26].

A natural polymer, such as collagen, which has a biological origin, is well suited for various in vivo applications. It promotes cell adhesion and growth by mimicking the key mechanobiological and biochemical features of the native ECM. However, most commercially available collagens are obtained from animal products such as tendons, skin, and bones, which creates anxiety around its quality, purity, disease transmission, and allergic response [27]. Alternatively, the development of molecular biology and gene engineering allowed researchers to produce a reliable and chemically defined recombinant human collagen peptide (RHC) without allergenicity [28,29]. For instance, the codon-optimized recombinant human collagen polypeptide monomeric gene has been designed and synthesized based on the mRNA sequence of type III human collagen. Then, the expression vector (pPIC9KG6) was transformed into *Pichia pastoris*. Subsequently, a high-level expression strain was selected from the transformants for high-cell-density fermentation [30]. The RHC-based biomaterials have been developed into porous scaffolds and hydrogels in our previous studies [31,32]. Therefore, RHC was chosen as the biomaterial to prepare nanofibers. However, scaffolds fabricated from collagen alone usually exhibit poor viscoelastic properties, break down quickly, and develop fast degradation rates and mechanical instability when uncrosslinked. On the other hand, PCL has a slower degradation rate, low cost, and distinct rheological and viscoelastic properties. Moreover, it serves a well-acknowledged role as a model biomaterial for specific long-term implantations [21,33]. Hence, composite scaffolds containing both RHC and PCL were made for nanofiber scale in this study.

Scaffolds with mechanical and structural properties close to those of the native vessels are desired in mimicking the autografts. Blood vessels have three distinct layers: intima, media, and adventitia that transport blood, oxygen, and nutrients to vital tissues and organs [34,35]. The intima consists of a layer of endothelial cells lining the vessels’ internal surface, which acts as an absorbent biopolymer membrane. Endothelial cells secrete soluble factors to maintain hemostasis and the anti-thrombogenic properties of the vessel walls. The media and adventitia contain smooth muscle cells and fibroblasts, and have a distinct circumferential orientation in order to provide the mechanical strength necessary to withstand the high pressures of the circulatory system [1,2]. Therefore, the ideal tubular scaffolds for vascular grafts may be bilayer grafts composed of a nanofiber membrane as the inner layer, and a porous layer with larger pores as the outer layer. Different research groups have attempted to fabricate bilayer and multilayer grafts [36,37,38].

Electrospinning-created grafts usually possess relatively small pores with low porosity, both of which limit cell infiltration. Many techniques have been used to improve pore diameters, such as utilizing salt [39], cryogenic electrospinning [40], increasing solution concentration, using scarifying polymers [41], or electrospinning with gas foaming [42,43], etc. In this study, sacrificial fiber was used with the co-electrospinning process in order to make the porous outer layer. Polyethylene oxide (PEO) is one of the best candidates for the formation of sacrificial fibers because of its high water solubility and adequate viscosity. Baker et al. [41] first introduced this combination of PCL and PEO fibers, where PEO fibers were sacrificed by dissolving them in water to produce PCL scaffolds with large pores and decreased fiber entanglement.

In this study, we hypothesized that pores of the outer vascular wall could be expanded by washing out PEO fibers to accommodate rat smooth muscle cells’ (A7r5) infiltration, while retaining a layer of smaller-diameter fibers near the lumen in order to facilitate adhesion of a continuous layer of human umbilical vein endothelial cells (HUVEC).

## 2. Materials and Methods

### 2.1. Materials

Recombinant human collagen peptides (Mw 112kD) with high hydrophilicity were prepared in our lab (The National Center for Biotechnology Information GenBank Access Number: EF376007). Polycaprolactones (PCLs) with Mn = 80,000 and 1,1,1,3,3,3-hexafluoro-2-propanol (HFIP) were purchased from Sigma (Shanghai, China). Polyethylene oxide (Mw 600kD) was purchased from Aldrich (Shanghai, China). 1-Ethyl-3-(3-dimethylaminopropyl) carbodiimide hydrochloride (EDC) was obtained from Yuanye (Shanghai, China). HUVEC and A7r5 cells were purchased from Chi scientific (Shanghai, China). Cell culture media and reagents were purchased from Hyclone (Shanghai, China). All other chemical reagents of analytical quality were obtained from Sinopharm Chemical Reagent (Shanghai, China).

### 2.2. Scaffold Fabrication

#### 2.2.1. The Inner Layer

**Figure 1:**
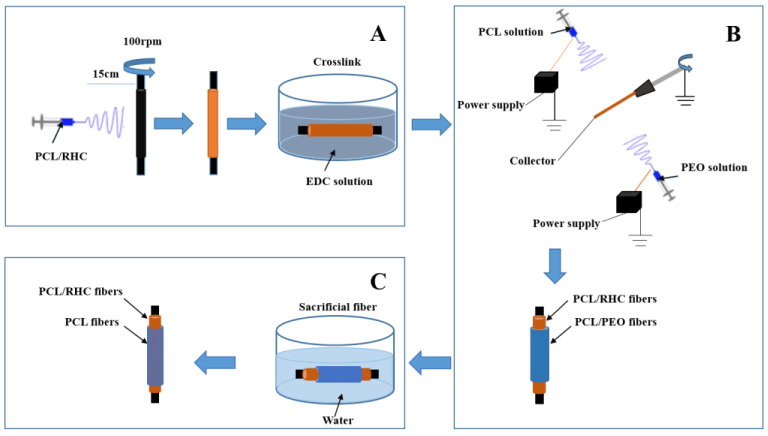
Schematic of the fabrication process of the bilayer scaffold: preparation of the inner layer (**A**), co-electrospinning of the outer layer of the scaffolds (**B**), removal of the PEO fibers (**C**).

In order to prepare the polymer solution for electrospinning the inner layer, PCL was dissolved in HFIP at 13% (*w*/*v*) and RHC 8% (*w*/*v*). After complete dissolution, PCL and RHC were mixed and stirred to prepare the blend solution with 4 ratios at 2:1, 1:1, 1:2, and 1:0, and the final bilayer scaffolds were named BS1 to 4, corresponding to the 4 different PCL/RHC ratios in this study. The electrospinning set-up included a syringe pump, a high-voltage supply, and a rotating mandrel. The blend solution was transferred to a 5 mL polypropylene plastic syringe with a 21 G stainless steel blunted needle at a constant flow rate of 0.4 mL/h using a syringe pump. A high voltage (20 kV) was applied to the polymer solution. The distance between the syringe tip and the mandrel was 15 cm and the rotation rate was approximately 100 rpm. Electrospun scaffolds were cross-linked in the 3 mM EDC, which was dissolved in a 90% ethanol solution at room temperature for 4 h. After being washed and hydrated in gradient ethanol, the internal fibers were freeze-dried (Figure 1A).

#### 2.2.2. Co-Electrospinning PCL/PEO (the Outer Layer)

A 13% *w*/*v* solution of PCL was prepared in HFIP. PEO was dissolved via stirring in 90% ethanol at room temperature for 4 h in order to yield a 5% *w*/*v* solution. The co-electrospinning PCL solution was fed into the first syringes (22 G), the PEO solution was fed into the other syringe (21 G), then both syringes were loaded on the pump. The needles were connected to a high voltage (20 kV) and at a working distance of 15 cm. The PCL solution was electrospun at a constant flow rate of 0.5 mL/h and the PEO solution at 2 mL/h. Collectors ran at a rotation speed of 100 rpm.

Co-electrospinning with a sacrificial layer of 5% *w*/*v* PEO on a rotating mandrel allowed for the fabrication of the bilayer scaffold (Figure 1B). After the co-electrospinning process, the scaffold was placed in deionized water for 1 d to dissolve the PEO fibers (Figure 1C). The final bilayer scaffold was freeze-dried for further use.

### 2.3. Morphology

The bilayer tubular graft’s structure and morphology were observed by SEM (Quant 250F, FEI, Hillsboro, OR, USA) at an accelerating voltage of 15 kV. Prior to SEM, the scaffolds were sputter-coated with gold to a thickness of 10–15 nm. In order to determine the average fiber diameter, SEM images were analyzed with Image-J (64-bit, National Institutes of Health, Bethesda, MD, USA). Average fiber diameter and distribution were calculated by randomly choosing 100 fibers from their respective SEM images (n = 100). As reported previously, porosity was calculated by using Photoshop (Adobe Systems, San Jose, CA, USA) and MATLAB (V9.7, MathWorks, Natick, MA, USA) [36]. Pore area was measured via subjective approximation of surface pores in the SEM images.

### 2.4. Mechanical Properties

#### 2.4.1. Axial and Circumferential Tensile

**Figure 2:**
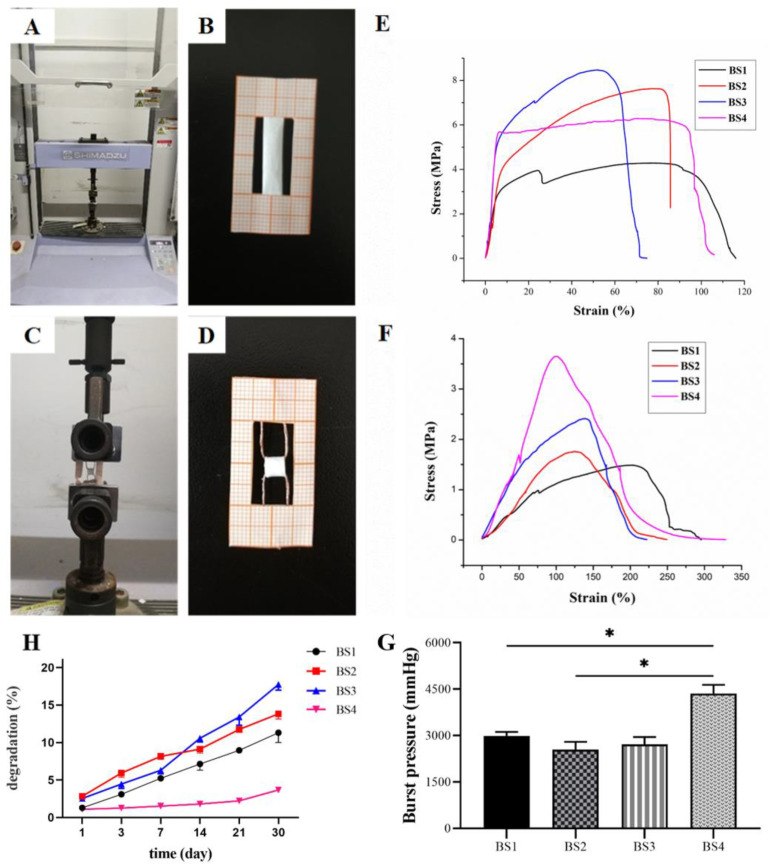
Universal testing machine (**A**), custom clamps for axial tensile tests (**B**) and circumferential tests (**C**,**D**), tensile stress–strain curves of four different bilayer scaffolds (BS1, BS2, BS3, BS4) by axial tests (**E**) and circumferential tests (**F**). Estimated burst pressure evaluated for four different bilayer scaffolds, * indicates the significance *p* ˂ 0.05 (**G**) and degradation rate in PBS solution (**H**).

Axial and circumferential tensile tests were conducted using a universal testing machine (Shimadzu AGS-X, Kyoto, Japan) in tension mode (Figure 2A). The thickness was measured using a micrometer caliper.

For axial tensile tests, tubular specimens (4 cm × 0.5 cm) (Figure 2B) were cut by a scalpel from different bilayer scaffolds. Samples were imbibed in phosphate-buffered saline (PBS) for 10 min, then tested with a cross-head speed of 2 mm/min until breaking.

Circumferential tensile tests were performed using home-made, ad hoc grips made of aluminum wires (diameter = 0.5 mm) (Figure 2C,D). Tubular specimens (0.4 cm × 0.5 cm) were cut by a scalpel from different scaffolds, imbibed in PBS for 10 min, then tested with a cross-head speed of 2 mm/min until breaking.

Ultimate tensile strength (UTS), Young’s Modulus, and elongation at breaking were obtained from the stress–strain curves (*n* = 5).

#### 2.4.2. Estimated Burst Pressure

In Gauvin et al.’s previous report [44], estimated burst pressure (BP) was calculated by rearranging Laplace’s law for a pressurized thin-walled hollow cylinder:

BP_estimated_ = 2 UTS.t/ID

where UTS was measured by circumferential tensile tests, t is the wall thickness (µm) of the scaffolds, and ID is the unpressurized inner diameter of the different tubular scaffolds (µm).

### 2.5. Degradation Test

The tubular specimen was cut by a scalpel from the different scaffolds into 2 cm × 0.5 cm pieces, then their weights were measured (W_0_). The degradation rate of various scaffolds was studied in vitro by degrading them in a PBS solution at 37 °C for 1 month. The buffer solution was renewed every 2 d. At predetermined time points (day 1, 3, 7, 14, 21, and 30), the specimens were removed from the buffer solution, rinsed at room temperature with deionized water, and freeze-dried. Weights were measured and set as W_t_. The degradation rate was determined using the following equation:

Degradation rate (%) = (W_0_ − W_t_)/W_0_ × 100

### 2.6. Swelling Ability

The swelling ability of the scaffolds was determined using the weighing method. Firstly, freeze-dried bilayer scaffolds were weighed (W_0_) and subsequently immersed in 37 °C PBS for 10 min. The soaked scaffolds were then taken out and any drops of PBS were carefully removed with filter paper before the wet bilayer scaffolds were weighed. This procedure was repeated until the weight of the wet scaffolds remained constant, and it was set as W_1_. The swelling ability of the bilayer scaffolds was defined by the following equation: Swelling degree (%) = (W_1_ − W_0_)/W_0_ × 100%.

### 2.7. Thermal Analysis

The thermogravimetric (TG) analysis of the bilayer scaffolds were determined by a TA Instruments sequential thermal analyzer (Model-SDT Q600, TA, New Castle, DE, USA). The analysis of the samples was tested from room temperature to 600 °C at a heating rate of 10 °C/min under nitrogen atmosphere.

### 2.8. Fourier Transform Infrared Spectroscopy (FTIR)

Chemical analysis of the optimization of the inner layer crosslinking was performed using Fourier transform infrared spectroscopy to qualitatively characterize the crosslinking. FTIR spectra were obtained using a Nicolet IS-10 FTIR Infrared Microscope (ThermoElectron Corporation, Waltham, MA, USA). All spectra were recorded in absorption mode at 2 cm^−1^ intervals and in the wavelength range of 4000–500 cm^−1^.

### 2.9. Scaffold Biocompatibility Assessments

#### 2.9.1. In Vitro Cell Culture

In order to evaluate the scaffolds’ responses, human umbilical vein endothelial cells (HUVEC) were cultured in F12 medium with 15% fetal bovine serum (FBS) and 1% antibiotics (100 IU/mL penicillin and 100 µg/mL streptomycin). A7r5 cells were cultured in DMEM medium with 15% FBS and 1% antibiotics. Fresh medium was replenished every 2 d, and the cultures were placed in 37 °C and 5% CO_2_, with a constant humidity of 95%. Before seeding, samples were disinfected via 70% ethanol vapor overnight, followed by rinsing with sterilized PBS solution. For each biocompatibility assessment, HUVEC was seeded on the inner layer while A7r5 was seeded on the outer layer. Tissue culture plates (TCP) were used for a control.

#### 2.9.2. Cell Proliferation Assay (CCK-8) and Morphology

For the proliferation evaluation, different samples were cut into the sizes of the 96-well plates. HUVEC proliferation was evaluated by a cell counting kit-8 (CCK-8) after the cells were cultured for 1, 3, and 7 d with a density of 5 × 10^3^ cells. At each time point, 10% CCK-8 was added and incubated for 2 h at 37 °C, aliquots were removed into a 96-well plate, and absorbance was measured at 450 nm by ELISA reader (Infinite 200 Pro, Tecan, Männedorf, Switzerland).

A7r5 cells were incubated in the same conditions as HUVEC, as mentioned above. A7r5 cells were seeded on the outer layer, TCP, and the control group with a density 3 × 10^3^ cells, then cultured for 1, 3, 7, 14, and 21 d. A7r5 proliferation was determined using CCK-8 at each time point.

In order to view the morphologies of the cells growing on various scaffolds, samples were stained with AO/EB (5 µL/mL each in PBS) at each time point. After staining for 3 min, samples were imaged at the WU module using a fluorescence microscope (Olympus IX81, Tokyo, Japan).

### 2.10. Statistics Analysis

Statistical analysis was completed using Origin 9.0 (Origin Lab Inc., Northampton, MA, USA). Experiments were performed in at least triplicates using the *t*-test, and the means were analyzed and expressed as mean ± standard deviation (SD). The differences in values were considered significant when *p* ˂ 0.05.

## 3. Results

### 3.1. Scaffold Morphological Characterization

**Figure 3:**
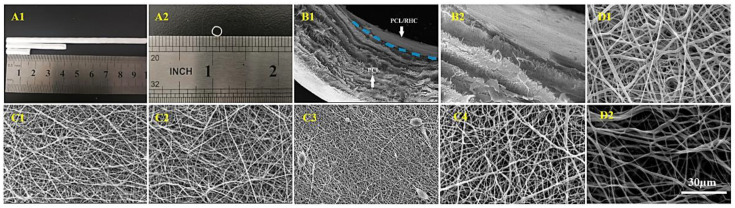
Digital images of scaffolds in the transverse (**A1**) and cross-section (**A2**). SEM images of a cross-section of the bilayer scaffold (**B1**) and a higher magnification image of the interface between both layers (**B2**). SEM images of electrospun internal layers (PCL/RHC) with different ratios: (**C1**) (2:1), (**C2**) (1:1), (**C3**) (1:2), and (**C4**) (1:0), as well as the outer layer (PCL/PEO) (**D1**), before washing out PEO and (**D2**): after washing out PEO.

SEM images in Figure 3 demonstrate structure and morphology of the electrospun fibers. Table 1 shows average fiber diameter, porosity, and pore area that were determined from SEM images by using Image-J software. The thickness of the bilayer structure was around 212.53 μm and the diameter of the scaffolds was found at 2.50 mm.

The inner layer was electrospun from a PCL/RHC polymer blend with four volume ratios, then crosslinked in the EDC solution at room temperature for 4 h. Average fiber diameter and porosity of 1:0 PCL/RHC layers (PCL pure) were 429.32 nm and 48.97% with random orientation (Figure 3(C4)). After adding RHC to the mixture with three ratios—2:1, 1:1, and 1:2—the average fiber diameter decreased while porosity increased. The 2:1 and 1:1 fibers had random distributions and exhibited smoothness (Figure 3(C1,C2)), and the average fiber diameter was around 299.49 nm for 2:1 and 324.52 nm for 1:1. The 1:2 fibers were random fibers and bead structures (Figure 3(C3)) with an average fiber diameter of 240.06 ± 66.12 nm and the highest porosity of 54%.

The outer layer was fabricated via co-electrospinning technique with sacrificial PEO fibers. The difference is indicated by images of two samples before and after washing out PEO (Figure 3(D1,D2)). For PCL/PEO, the average fiber diameter was 852 nm, porosity was 44.29%, and pore area was 7.76 µm^2^. After washing out PEO, the average fiber diameter increased to 1189.8 ± 417.7 nm, the porosity increased to 76.85%, and the pore area increased to 27.9 µm^2^.

### 3.2. Bilayer Scaffold Mechanical Properties

Mechanical properties were evaluated with four bilayer scaffolds (BS1, BS2, BS3, BS4), as distinguished by the ratio of their inner layer (PCL/RHC ratio) construction. Table 2 demonstrates the ultimate tensile strength, strain at breaking, Young’s modulus, and calculated burst pressure of bilayer scaffolds with the axial tensile test and the circumferential test. The representative tensile stress–strain curves of bilayer scaffolds by axial tests and curves of circumferential tests are shown in Figure 2E,F.

#### 3.2.1. Tensile Axial Test

Axial tensile properties showed that the four bilayer scaffolds had good mechanical properties. Table 2 illustrates that all four bilayer scaffolds had mechanical compatibility relative to native vasculature. The Young’s modulus values of the four bilayer scaffolds were 4.08, 2.19, 2.16, and 4.92 MPa, respectively. The strain at breaking of the four bilayer scaffolds far exceeded the native vasculature, in which the strain at breaking of BS1 was highest up to 116%.

#### 3.2.2. Circumferential Tensile Test

The circumferential tensile of the bilayer scaffolds are shown in Table 2. BS1 had the lowest ultimate tensile strength (1.48 MPa) under the circumferential test, and the strength increased with the increasing of the RHC content, while the PCL-only scaffold had the highest ultimate tensile strength (3.65 MPa). Meanwhile, the BS1 had the highest strain at break among all RHC-containing groups, and the PCL-only scaffold (BS4) had a strain at break of 328.3%.

#### 3.2.3. Estimation of Burst Pressure

Burst pressure was estimated from the stress/strain curves obtained in the tensile circumferential tests. A significant difference (*p* ˂ 0.001, Figure 2G) was observed in the burst pressure of the four bilayer scaffolds, with BS4 having the highest burst pressure (4356 mmHg) compared to the other scaffolds, and there were no significant differences found among BS1 to 3. 

### 3.3. Degradation Tests

A crucial part of new tissue regeneration is tissue scaffolds with appropriate degradation rates. The degradation rates of four different bilayer scaffolds were measured in a PBS solution at 37 °C for 30 d. Figure 2H shows the weight loss of four bilayer scaffolds at each time point. After 30 d of degradation, BS3’s weight loss was the highest at approximately 17.4%, BS2’s weight loss was about 13.8%, BS1’s weight loss was about 11.3%, while BS4 had the slowest degradation rate, which was around 3.7%. 

### 3.4. Swelling Ability

**Figure 4:**
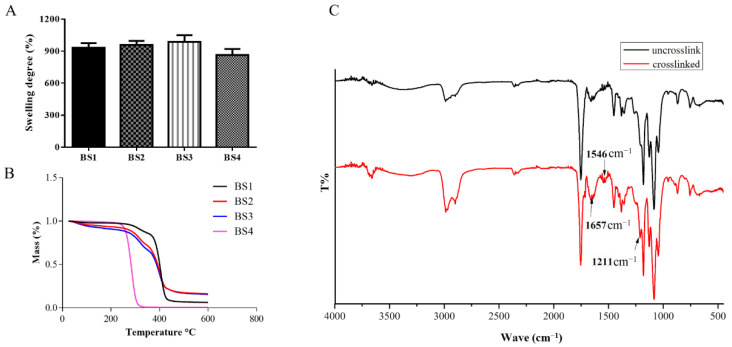
Swelling ability of bilayer scaffolds (**A**), TG analysis of bilayer scaffolds (**B**), FTIR spectra obtained for PCL/RHC inner layer samples that were uncrosslink and crosslinked (**C**).

The swelling ability of the bilayer scaffolds were measured by soaking the samples in 37 °C PBS. Figure 4A shows that BS3 had the largest swelling degree, which was approximately 989%, while swelling degrees of BS1 and BS2 were found to be 933% and 959%, respectively. The swelling degree decreased to 865% in the BS4 group.

### 3.5. Thermal Analysis

The thermal stability of bilayer scaffolds was analyzed by TG (Figure 4B). The first stage of weight loss on BS1, BS2, and BS3 samples took place at around 50 to 210 °C, where 3% to 10% weight loss was observed in this stage. The weight loss of BS4 was found from 210 to 310 °C, and its weight loss reached approximately 95%. The second stage of weight loss for BS1, BS2, and BS3 samples was located around 270 to 415 °C, where the weight loss ranged 85 to 94%.

### 3.6. FTIR Spectroscopy

The structures of the test samples were determined by FTIR, as shown in Figure 4C. Band positions and shapes could provide information about the molecular structures and changes that follow the cross-linking process. The RHC structures of inner layers (PCL/RHC) of the scaffolds were determined in this study. Typical bands of collagen, including CH_2_ (2987 cm^−1^), Amide A (2990 cm^−1^), Amide B (2980 cm^−1^), Amide I (1657 cm^−1^), Amide II (1546 cm^−1^), and Amide III (1211 cm^−1^), were observed in both spectrums. The intensity of Amide I, Amide II, Amide III, Amide A, and Amide B were found increase after crosslinking, while no shifting of these bands was observed between the two samples.

### 3.7. Viability of Cells

#### 3.7.1. Endothelial Test

**Figure 5:**
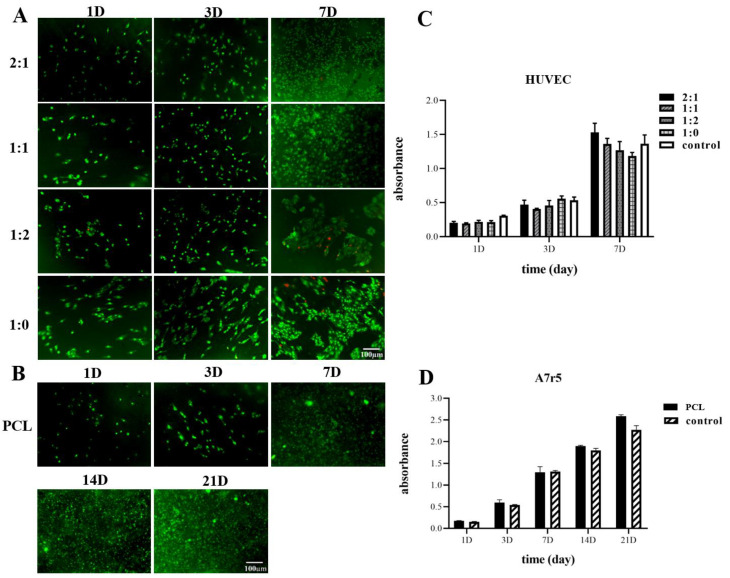
Morphology and proliferation evaluations of cells on different scaffolds. (**A**) AO/EB staining of HUVEC cells grown on four different scaffolds. (**B**) AO/EB staining of A7r5 grown on the outer layer. (**C**) Viability of HUVEC cells measured by the CCK-8 assay. (**D**) Viability of A7r5 measured by the CCK-8 assay.

Endothelial cells were seeded into four different scaffolds in order to compare the biocompatibility of the four inner layer samples (Figure 5A). AO/EB double fluorescent staining was conducted to further investigate the morphology and growth status of the cells, with the living cells emerging as bright green, the apoptotic cells bright orange, and the dead cells bright red. On the first day, the cells were distributed randomly and exhibited round, green shapes. Cell density increased at each time point for all four scaffolds, and the membranes were fully covered with proliferated cells on day 7. The proliferation rates of the seeded HUVEC were determined by CCK-8 assay, where cells grown in tissue culture plates (TCP) were set as control (Figure 5C). The CCK-8 results indicated that, after 7 d of culturing, the number of cells increased at three time points. Likewise, the number of cells on the PCL-only scaffold was similar as compared to the PCL/RHC scaffold and the control. The number of cells on the three PCL/RHC scaffolds increased compared to the PCL pure scaffold, and the 2:1 ratio scaffold had the highest number of cells, even higher than that of the control.

#### 3.7.2. A7r5 Test

In order to evaluate the proliferation and infiltration of A7r5 on the outer layer, A7r5 cells were cultured for 3 weeks in the PCL/PEO scaffold washed out PEO fibers (Figure 5B). Figure 5D shows cell viabilities measured at five time points by CCK-8 assay with cells grown in a TCP set as the control. After 3 weeks, cells proliferated through the scaffold even better than TCP, since PCL/PEO scaffold washed out PEO fibers have a microfibrous structure with a high porosity and large pore area for the cells to attach and grow. Figure 5B shows AO/EB staining results, indicating that A7r5 cells were well proliferated and infiltrated into the outer layer within the culturing time. 

## 4. Discussion

Tissue engineering offers a viable approach to vascular grafting and a common approach for imitating the structural and mechanical properties of native blood vessels. The materials used in fabricating the artificial blood vessels are also quite biocompatible, which provides a favorable environment for the growth of vascular cells, the same as the process that occurs with a real body. Autografts, such as the saphenous vein and the internal mammary artery, have a layered structured, wherein each layer serves a specific function to provide cell guidance and attachment sites, while the elastic lamina provides the vessels walls with elasticity and functions as structural support for cells [8]. Thus, we used an electrospinning technique combined with freeze-drying to fabricate a bilayer scaffold to mimic the native blood vessels.

The mechanical properties of the bilayer graft are also essential in biomimetic native blood vessels. In previous reports, S. K. Norouzi [38] showed that bilayer heparinized vascular graft has a Young’s modulus of 1.55 + 0.32 MPa, which is close to the coronary artery and burst pressure of 882 + 56 mmHg and less than burst pressure of the widely used autologous graft, the saphenous vein, but large enough to sustain physiological circumstances. In the current study, all four bilayer grafts had Young’s modulus values comparable to that of a coronary artery (1.41 ± 0.72 MPa) [45], and the axial and circumferential strains at break of the four bilayer scaffolds exceeded the native saphenous and femoral artery (11% and 63–76%, respectively) [46], results that are comparable to the mammary arteries (134%) and the saphenous vein (180–242%) [47]. For tubular grafts carrying pressurized blood, burst pressure is an important parameter. The tested results illustrated that adding of RHC lead to a decrease in the burst pressure, which corresponded to results of the tensile test. PCL/RHC scaffolds in this study had burst pressures that are less than that of mammary arteries (3196 ± 1264 mmHg), but higher than that of saphenous veins (1599 ± 877 mmHg) [47]. This demonstrates that it can function well even in high-blood-pressure conditions.

PCL is a synthetic polymer that degrades slowly over several months, while collagen is an amorphous polymer. In a hydrolytic degradation of a scaffold, the crystalline region degraded more slowly than the amorphous region [48], and the biodegradable process of PCL takes much longer than collagen. Q. Zhang et al. [49] showed that after immersion for 21 days in PBS solution, the weight loss of PCL/Col scaffold was found to only reach 2.21–2.97%. To overcome the fast degradation of collagen in vivo, the collagen hydrogel should be crosslinked chemically. In this study, the inner layer was reinforced by cross-linking in EDC solution and the degradation rates of bilayer blood vessels were found to increase with the addition of RHC. After immersion for 30 days in PBS solution BS1′s weight loss was around 11.3%. All porous bilayer scaffolds showed great liquid-maintaining ability in a current test, and the swelling ratio of the bilayer scaffolds were found to be RHC dependent, indicated by the fact that by increasing the ratio of RHC, the swelling degree slightly increased. This phenomenon might be due to the hydrophilic groups present on the RHC molecules, while another possible explanation is that the RHC-containing scaffolds had a larger pore size as they could maintain more liquid. In the thermal stability study, the first stage of weight loss of BS1, BS2, and BS3 was mainly due to the evaporation of physisorbed water within the samples. Water can be bound in both hydroxyls and amines of the RHC molecules [50], thus, all RHC-containing samples went through this stage, while BS4 fabricated from pure PCL showed different behavior. The weight loss stage (210–310 °C) of BS4 illustrated the decomposition of the PCL and second stage (270–415 °C) of weight loss on BS1, BS2, and BS3 samples was associated with the decomposition of the collagen molecules [51]. By adding RHC and chemically crosslinking the sample with EDC, it was found that their weight loss stages shifted to a higher temperature, which indicated the thermal stabilities of the bilayer scaffolds increased when compared to BS4. The FTIR results suggested that EDC as efficient chemical cross-linker increased the number of peptides in RHC molecules without affecting the secondary structure of the collagen, thus, the degradation rate of the fabricated nanofibers was decreased.

HUVEC cells play important roles in tissue homeostasis, fibrinolysis, blood cell activation, and anti-coagulation during both physiological and pathological processes. Achieving a uniform endothelium requires HUVEC adhesion and proliferation on the lumenal surface of the scaffold, therefore, it should cover the scaffold’s entire luminal surface. AO/EB staining results on day 7 indicated that the biocompatibility rates of the PCL/RHC scaffolds were better than that of the PCL-only scaffold. Most of the HUVEC cells presented a green elliptical morphology fully covering the PCL/RHC scaffold, while these appeared as a few dead cells on the PCL pure scaffold. This study also compared four samples in order to evaluate how HUVEC attached and proliferated by using CCK-8 assay. Results indicated that the PCL/RHC (2:1) scaffold with fiber diameter 299.49 nm (Figure 3(C1)) had the highest proliferation rate among all tested samples. The 1:2 ratio scaffold, also showed a few dead cells, which might explain the impact of the degradation rate on biological compatibility. A7r5 cells are known to perform multiple functions, including vasoconstriction and dilatation; various types of collagen, elastin, and proteoglycans syntheses; and the elaboration of growth factors and cytokines [52]. A7r5 cells should be homogeneously distributed within the outer portion of the scaffold before transplantation. Therefore, the outer layer with porous structures and ideal pore size could promote the A7r5 infiltration easily. We used co-electrospinning PCL/PEO with sacrificial PEO to prepare the porous layer with a pore area of 27.9 µm^2^ and a porosity up to 76.85% (Figure 3(D2)), and in vitro results illustrated that the A7r5 cells could proliferate on the porous layer of the scaffolds. Meanwhile, these cells were found to be capable of penetrating the porous layer by increasing the culturing time. These results demonstrated that when RHC was added to scaffolding, the biocompatibility increased, cell adhesion improved, and cell proliferation improved, which is similar to previous reports [53]. RHC’s excellent hydrophilicity contributed to the improved cell viability on nanofiber membranes, which facilitates the formation of the lumen.

## 5. Conclusions

To model the multilayer morphology of native vasculature, a bilayered scaffold with fibrous inner layer and porous outer layer was developed by using electrospinning the RHC and PCL. The inner fiber layer were fabricated by electrospinning PCL/RHC (2:1) polymer solutions, which allow HUVEC adhesion onto the lumenal surface. As well, a predefined porous outer layer was fabricated by co-electrospinning PCL and PEO solutions with sacrificial PEO fibers, which allow homogenous infiltration of A7r5 into this layer. Results demonstrated the scaffolds are biocompatible, biodegradable, easily fabricated, and able to support cell adhesion and infiltration. Moreover, the bilayer graft shows adequate mechanical properties that withstand physiologically relevant vascular conditions. Thus, it offers a promising approach for further in vivo studies to fully assess the performance and mechanical properties of this scaffold as potential prosthetic vasculature.

## Figures and Tables

**Table 1:** Fiber diameter, porosity, and pore area of different electrospun membranes.

	PCL/RHC				PCL/PEO	
	BS1	BS2	BS3	BS4	Non-Washing Out PEO	Washing Out PEO
Fiber diameter (nm)	299.49 ± 91.6	324.5 ± 97.3	240 ± 66.1	429.3 ± 17.6	852 ± 320	1189.8 ± 417.7
Porosity (%)	51.62 ± 1.13	52.51 ± 1.08	54 ± 2.75	48.97 ± 1.02	44.29 ± 0.84	76.85 ± 2.97
Pore area (µm^2^)	-	-	-	-	7.76 ± 0.64	27.9 ± 1.15

**Table 2:** Mean and standard deviation values of the mechanical parameters obtained by axial and circumferential tensile tests for four different bilayer scaffolds (BS1, BS2, BS3, and BS4).

	Bilayer Scaffold	BS1	BS2	BS3	BS4
Axial tensile test	Ultimate tensile strength (MPa)	4.28 ± 0.21	7.63 ± 0.81	8.47 ± 0.6	6.16 ± 0.74
	Strain at break (%)	116.07 ± 7.49	85.7 ± 3.49	74.83 ± 2.5	105.97 ± 4.8
	Young’s Modulus (MPa)	4.08 ± 0.75	2.19 ± 0.2	2.16 ± 0.18	4.92 ± 0.63
Circumferential tensile test	Ultimate tensile strength (MPa)	1.48 ± 0.05	1.74 ± 0.14	2.40 ± 0.19	3.65 ± 0.2
	Strain at break (%)	295.6 ± 10.78	249.3 ± 16.1	222.3 ± 12.5	328.3 ± 19.1
	Young’s Modulus (MPa)	0.53 ± 0.04	0.59 ± 0.06	0.78 ± 0.05	0.64 ± 0.08

## Data Availability

Not applicable.
